# The interplay between task difficulty and microsaccade rate: Evidence for the critical role of visual load

**DOI:** 10.16910/jemr.13.5.6

**Published:** 2021-04-28

**Authors:** Andrea Schneider, Andreas Sonderegger, Eva Krueger, Quentin Meteier, Patrick Luethold, Alain Chavaillaz

**Affiliations:** University of Bern, Switzerland; École Polytechnique Fédérale de Lausanne (EFPL), Switzerland; Bern University of Applied Scienes, Switzerland; University of Fribourg, Switzerland

**Keywords:** Fixational eye movements, microsaccades, visual load, mental load, measurement, eye behavior, eye tracking

## Abstract

In previous research, microsaccades have been suggested as psychophysiological indicators of task load. So far, it is still under debate how different types of task demands are influencing microsaccade rate. This piece of research examines the relation between visual load, mental load and microsaccade rate. Fourteen participants carried out a continuous performance task (n-back), in which visual (letters vs. abstract figures) and mental task load (1-back to 4-back) were manipulated as within-subjects variables. Eye tracking data, performance data as well as subjective workload were recorded. Data analysis revealed an increased level of microsaccade rate for stimuli of high visual demand (i.e. abstract figures), while mental demand (n-back-level) did not modulate microsaccade rate. In conclusion, the present results suggest that microsaccade rate reflects visual load of a task rather than its mental load.

## Introduction

Research on microsaccades has been very popular in the past decade (1, 2), with a considerable deal of effort being put into investigating causes for the modulation of microsaccade rate (3, 4). In this context, the influence of task load on microsaccade rate attracted considerable interest in research (5, 6, 7, 8, 9). However, the result pattern of studies addressing the relation of microsaccade rate and task load is rather diverse and complex. While it can be assumed that task difficulty has a decisive influence on microsaccade rate (5, 6, 7, 8, 10, 11, 12), the direction of this effect is less apparent. Some studies indicate an increase while others report a decrease in microsaccade rate as consequence of an increase in task difficulty. In this context, visual load, i.e. the amount of information involved in the perceptual processing of the task stimuli (see Lavie (13) for review), and/or mental load, i.e. demands of working memory (14) have been suggested to play a major role (12). Although results of some initial studies comparing different types of cognitive load (e.g. 9, 12) indicate that the visual component of the load is highly important for microsaccade occurrence, there is still some lack of clarity regarding the influence of non-visual task load.

### Microsaccades and task difficulty

Microsaccades are small saccades which are produced when attempting to fixate the gaze on a visual target. They contribute to maintaining visibility during fixation by shifting the retinal image to overcome adaption (15, 16). Research has shown that microsaccades serve not only oculomotor functions but can also be modulated by attention (17, 18).

There seems to be a consensus that microsaccade rate is modulated by both endogenous attentional shifts (i.e. top-down mechanisms voluntarily driving attention) and exogenous attentional shifts (i.e. reflexive or bottom-up mechanisms drawing attention automatically towards a stimulus, (17, 19, 20, 21). In later studies, a link between microsaccade production and other cognitive processes such as working memory was made (3, 4, 21, 22).

Recent studies showed differing patterns of microsaccadic activity in relation to applied tasks, depending on task modality (visual task, e.g. visual search task, or mental task, e.g. arithmetic task) and the variation of difficulty within those tasks (6, 7, 8, 11). Some research revealed a positive correlation between microsaccade rate and task demand. For instance, Benedetto and colleagues (11) used a simulated driving task to compare a low load task (control task) and a high load task (dual task including visual search task) and reported more microsaccades being produced in the high load condition. However, other studies reported lower microsaccade rate associated with an increase of mental load. Those studies applied non-visual tasks (6, 7, 8). For example, Siegenthaler and colleagues (6) used a mental arithmetic task without any visual component (mental counting) and reported that microsaccade rate decreased in the high load condition. A replication of Siegenthaler et al. (6) showed the same inverse relationship between microsaccade rate and task difficulty (7). Dalmaso and colleagues (8) applied two-digit (low load) and five-digit (high load) memorizing tasks to investigate the association between microsaccade rate and working memory load. Their results indicated also a reduced microsaccade rate for the task with high mental load.

Overall, studies have shown that increasing difficulty in tasks with a strong but not exclusive visual component increases microsaccade rate (11) and that increased task difficulty in non-visual tasks is linked with a decreased microsaccade rate (6, 7, 8). These contradicting results indicate that specific interactions between the effects of visual load and mental load might occur.

To learn more about those interactions, Krueger and colleagues (9) manipulated visual and mental load systematically in a dual-task paradigm (visual search task and mental arithmetic task). They showed that if processing resources are allocated to a visual task (i.e. a difficult visual task combined with an easy mental task), microsaccade rate increased with difficulty level. On the other hand, microsaccade rate decreased with increasing difficulty level of the mental task (i.e. a difficult mental task combined with an easy visual task). The authors hence concluded that microsaccade rate indicates how much processing resources are allocated to a visual task.

In summary, previous research has shown that task load modulates microsaccade rate; visual load is associated with an increase in microsaccade rate while mental load is associated with a decrease in microsaccade rate (9). But it is not clear yet whether inducing visual or mental load is decisive for changing microsaccade rates.

Therefore, the intriguing remaining open question is how the microsaccade rate is modulated when mental load is induced by a task that requires visual information processing. Or put differently, the question is whether the microsaccade rate responds to an increase in difficulty of mental load in a task where visual information processing is required to solve the task.

### The present study

In contrast to all previous research, the task used in this study coupled visual and mental demand. This means that some degree of visual processing must be maintained during the entire task. Processing resources have thus to be split between the visual and the mental processing. For this purpose, two versions of a n-back task were created, to manipulate visual difficulty of the task. The figure version used stimuli that are novel, visually complex and difficult to process in comparison to the letter version using well-known stimuli that are easy to process. Mental load was manipulated by increasing difficulty level (increasing n).

Based on previous studies we expected 1) higher microsaccade rate in the condition inducing higher visual load (n-back task with figures) compared to the task inducing a minimal amount of visual load (n-back task with letters), and 2) microsaccade rate to decrease with increasing mental load.

## Method

### Participants

Fourteen participants (55% females) with an average age of 21.45 years (SD ± 1.29) took part in the study. All participants were students of the University of Fribourg. Three participants were excluded from the eye movement analyses due to technical problems.

### Task and Stimuli

Participants completed two versions of the n-back task. The n-back tasks are continuous recognition measures that present sequences of stimuli, such as letters or pictures (originally introduced by Kirchner (23)). For each item in a sequence, participants judge whether it matches the one presented n items ago. n is a variable that can be adjusted to respectively increase or decrease mental load (24). In the current study, we used two different sets of stimuli, letters and Attnaeve figures (25, 26). The presentation of unknown figures is associated with an increase in visual load (as compared to well-known stimuli like letters). In addition, participants cannot use verbal strategies when Attnaeve figures are presented.

In addition, a probe detection task (PDT) was used as a control task. In this task, participants were required to identify a predefined stimulus (a star) among distractors (a diamond).

**Figure 1.**
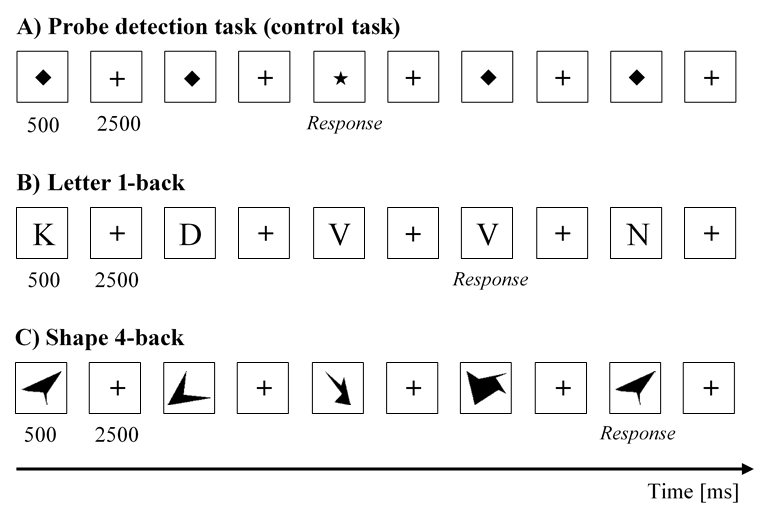
Illustration of the probe detection task (control task) (A), the letter version (B), and shape version (C) of the n-back task.

**Figure 2.**
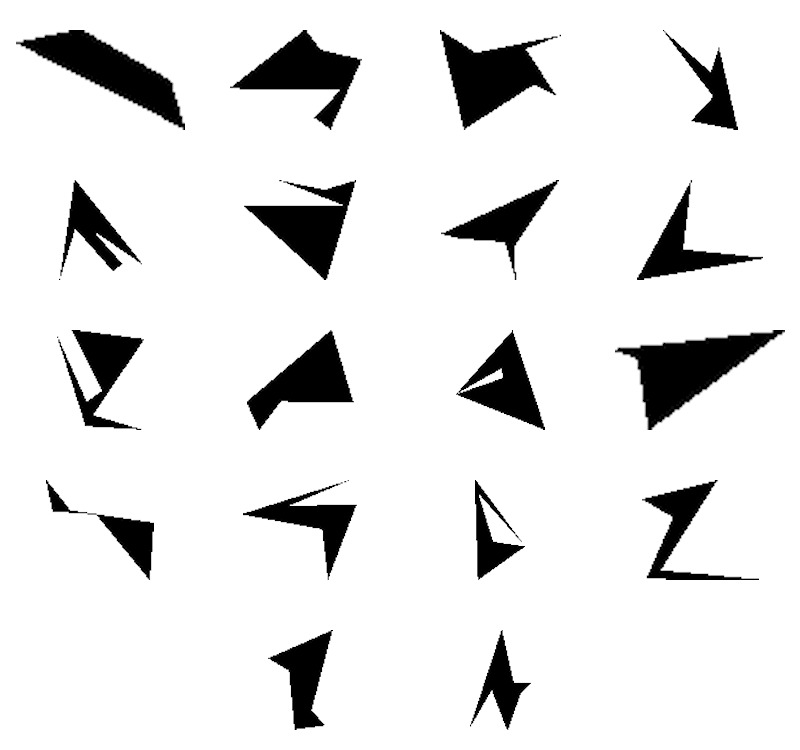
Entire set of shape stimuli.

Each version of the n-back task contained eight trials. Starting with 1-back, it increased up to 4-back and reversed. Furthermore, each version started and ended with a probe detection task, for a total of 10 trials (control, 1-back, 2-back, 3-back, 4-back, 4-back, 3-back, 2-back, 1-back and control). Eighteen stimuli appeared in each trial. Each stimulus was presented for 500ms, followed by a black fixation cross for 2500ms (see Figure 1). Six target stimuli appeared in each trial at random positions. Participants were asked to press the spacebar as fast as possible when they detected a target and do nothing when a distractor was displayed. To avoid any predictability in the n-back tasks, the stimuli sets were divided into three groups which served an equal amount of times as targets and distractors across observers. This means that each stimulus was a target in about a third of the trials, a distractor in another third and was not present in the last third of the trials. The use of each stimulus (target, distractor or not used) was counterbalanced across trials and participants. The stimuli set in the letter condition consisted of 18 capital consonants (B, C, D, F, G, H, J, K, M, N, P, Q, R, S, T, V, X, Z, e.g. Ravizza et al., 2004). Meaningless Attneave figures composed the figure stimuli set to ensure that observers could not remember the figures due to their meaning. They had between four and eight sides and were selected among about 200 figures randomly generated with the Matlab® code developed by Collin and McMullen (27) (see Figure 2). They were selected based on the work of Vanderplas and Garvin (28). Letter and figure stimuli were about the same size (height of 2.33° by width of 2.37° of visual angle, with a standard deviation of 0° and 0.77°, respectively). The presentation order of the n-back versions were counterbalanced across participants.

### Experimental design

In this study, a 2 x 10 within-subject design was used, with visual load and difficulty order as factors. Visual load was manipulated by using different types of visual stimuli for the n-back task on two levels, letters (i.e. easily processed stimuli) and Attneave figures (i.e. hard-to-process stimuli (25, 26)). Difficulty order corresponded to the sequence in which trials were presented according to their difficulty.

### Apparatus

The experiment was conducted in a quiet room, which was equally illuminated for each session. Participants were seated at a desk approximately 70cm away from an LCD monitor (1650 by 1050 px, 60 Hz refresh rate), facing a desktop-mounted EyeLink 1000 eye tracker. Head position was maintained constant by an EyeLink 1000 head/chin support. Stimuli presentation and data collection was controlled by a Matlab script and Psychophysics and EyeLink Toolbox extensions ((29, 30, 31); see http://psychtoolbox.org/).

### Measures

Microsaccades per second and response accuracy were recorded. Response accuracy (i.e. percentage of correct responses) was used as performance measures.

In addition, participants completed a modified NASA-Task Load Index (NASA-TLX; (32, 33)) for mental demand (How mentally demanding was the task?) and effort (How hard did you have to work to accomplish your level of performance?), as a measure of perceived workload. A visual analog scale ranging from 0 to 100 with a title and a bipolar descriptor (very low/very high) at each end was presented for both NASA-TLX dimensions.

### Eye movement recordings and analyses

A non-invasive fast video-based eye tracker (Eyelink 1000, SR research, Ontario, Canada) sampling the eye position binocularly at a frequency of 500Hz was used to assess eye movement data. Blinks and semiblinks were removed for data analysis. Blinks correspond to a full occlusion of the pupil and were identified by the missing information about the pupil. Semiblinks are periods with very fast changes of the pupil area during which the pupil is never completely occluded. A decrease or increase larger than 50 pupil size units per sample was considered as a semiblink (20). Additionally, to avoid any partial occlusion, data samples 200ms before and after blinks and semiblinks periods were removed (20). Microsaccades were automatically identified with an objective algorithm (see Engbert & Kliegl (17) for details) using a λ of 6 to determine the velocity threshold and a selection criterion of an amplitude smaller than one degrees of visual angle (c.f. also 3, 20, 34, 35) based on the entire trial. The minimum duration for a microsaccade was set at 10ms. To reduce potential noise (1, 36), only binocular microssacades were retained for the analyses (e.g. 1, 20, 37). To avoid categorizing overshoots as microsaccades, an intersaccadic interval of 20ms was applied (17).

### Procedure

Participants were welcomed in the eye tracking laboratory and guided to their place. The experimenter explained the procedure as well as the tasks and asked for the following information about the participant: gender, age, and handedness. Then, participants were seated at a desk in front of the LCD monitor. Participants performed both versions of the n-back task which differed in type of stimuli presented (letters or Attnaeve figures). For each version, a nine-point calibration of the eye position was performed before completing the ten experimental trials. Furthermore, to maintain the accuracy of the eye position signal, a drift correction was performed at the beginning of each trial. Finally, they had to respond to the two questions about their subjective state at the end of each trial.

### Data analysis

Data were analysed with a two factorial repeated measures ANOVA with stimuli type (two levels: letters and Attneave figures) and task difficulty (5 levels, control task to 4-back) as within-subjects factors with performance and mental workload as dependent variables. A second two factorial repeated measures ANOVA with stimuli type (two levels: letters and Attneave figures), task difficulty (five levels, control task to 4-back) as within-subjects factors was calculated to analyze the effect of stimuli type, task difficulty and object processing on microsaccade rate. Furthermore, a 2 (letters, Attneave figures) x 8 (task difficulty from 1-back to 4-back and from 4-back to 1-back) x 2 (displayed stimulus: fixation cross and stimulus) repeated measures ANOVA was also used to investigate how the microsaccade rate behavior was affected by the stimuli type, variations of task difficulty across trials and the relevance of the displayed object for the task. Partial eta-squared (*η_p_^2^*) was calculated as effect size measure.

Finally, we investigated whether microsaccade rate was influenced by expected response to the stimulus (target vs distractor). We used a similar approach as described in Engbert & Kliegl (17) where microsaccade rate was averaged overall all trials of all participants for each level of task difficulty. A moving time-window of 100ms was used to smooth the data.

## Results

### Performance and subjective workload

**Figure 3.**
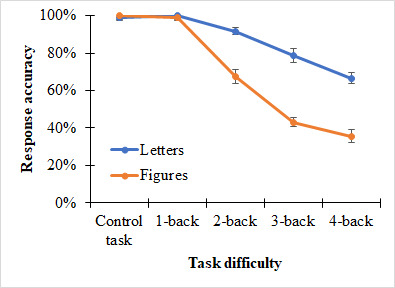
Means and standard errors for performance (i.e. response accuracy) as a function of task difficulty (mental load) and stimulus type (visual load). Data of the respective two levels of mental load (e.g. the two 1-back trials with letter stimuli) were pooled.

Analysis of performance data (see Figure 3) indicates reduced performance when the Attnaeve figures were used compared to the letter stimuli, *F*(1, 10) = 56.21, *p* < .001, *η_p_^2^* = 0.85. In addition, performance decreased with an increase in task difficulty, *F*(2.56, 25.58) = 63.66.0, *p* < .001, *η_p_^2^* = 0.86, with within-subjects contrasts revealing a significant linear trend, *F*(1, 10) = 150.78.0, *p* < .001, *np^2^* = 0.85. Also the interaction of stimulus type and n-back level reached significance level, *F*(2.50,25.02) = 6.00, *p* < .001, *η_p_^2^* = 0.71. Accuracy was similar in the control and 1-back conditions for both visual load conditions but decreased faster in for figures than letters from the 2-back to the 4-back condition.

**Figure 4.**
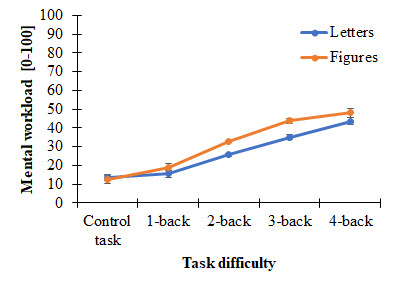
Means and standard errors for perceived workload as a function of task difficulty (mental load) and stimulus type (visual load). Data of the respective two levels of mental load (e.g. the two 1-back trials with letter stimuli) were pooled

The reverse pattern was observed for perceived workload (see Figure 4). Subjective workload was higher with Attneave figures than with letters, *F*(1, 10) = 51.89, *p* < .001, *η_p_^2^* = 0.84.

Furthermore, it increased according to task difficulty, *F*(1.16, 11.64) = 57.77, *p* < .001, *np^2^* = 0.85, in a linear way, *F*(1, 10) = 64.08, *p* < .001, *np^2^* = 0.84. Finally, this increase was steeper for the figures compared to the letters stimuli, as revealed by the significant interaction effect *F*(2.22, 22.19) = 9.30, *p* < .001, *np^2^* = 0.48. Altogether, these results indicate that the experimental manipulation was successful.

### Microsaccade rate

The 2x5 repeated measures ANOVA revealed a significant main effect of stimulus type on microsaccade rate, *F*(1, 10) = 50.80, *p <* .001, *η_p_^2^* = 0.84. Microsaccade rate per second in the figure condition was higher (*M* = 1.74, *SD* ± 0.46) compared to the letter condition (*M* = 1.48, *SD* ± 0.46).

Regarding the task difficulty level, results indicated a significant main effect on microsaccade rate, *F*(1.72, 17.22) = 8.00, *p = .*005, *η_p_^2^* = 0.44. Simple contrasts with the control condition as reference revealed that microsaccade rate differed significantly between the ‘control task‘ and all other condition, *F*s > 7, *p* < .05, expect for the 2-back condition, *F*(1, 10) = 2.34, p = .157, *η_p_^2^* = .19.

The interaction between stimulus type and mental demand level was significant, *F*(4, 40) = 11.18, *p* = .007, *η_p_^2^* = 0.53. As displayed in Table 1, further analyses revealed that the microsaccade rate was significantly higher for figure stimuli compared to the letter stimuli in each mental load condition except for the control condition.

**Table 1.** Descriptive data (mean and standard deviation) and *t*-test results (*t*-value, one-tailed significance level and effect size) of microsaccade rate per second as a function of stimulus type (visual load).

Task difficulty	Letters *M* (*SD*)	Figures *M* (*SD*)	*t*(10)	*p*	*ηp²*
Control	1.41 (0.44)	1.39 (0.59)	00.173	.866	0.055
1-back	1.46 (0.53)	1.86 (0.42)	-4.776	.001	0.834
2-back	1.42 (0.50)	1.67 (0.51)	-2.461	.034	0.614
3-back	1.52 (0.50)	1.82 (0.50)	-3.831	.003	0.771
4-back	1.58 (0.48)	2.00 (0.47)	-8.068	.000	0.931

**Figure 5.**
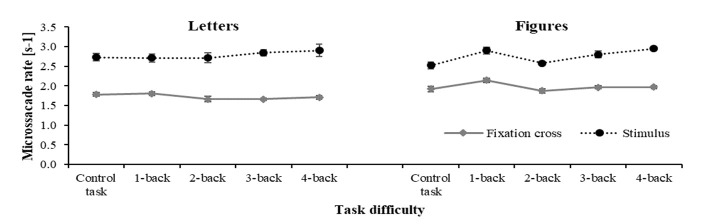
Means and standard errors for microsaccade rate per second as a function of n-back level (mental load) and displayed stimulus (fixation cross vs stimulus) for each stimulus type (visual load).

As expected from the previous analysis, the 2x8x2 repeated measures ANOVA confirmed a higher microsaccade rate in the figure condition (*M* = 1.93, *SD* ± 0.59) than in the letter condition (*M* = 2.92, *SD* ± 0.37), *F*(1, 8) = 21.972, *p = .*002, *η_p_^2^* = 0.733 (see Figure 5). Furthermore, there was no difference between blocks, *F*(1, 8) = 1.902, *p = .*086, *η_p_^2^* = 0.192, nor any interaction between stimulus type and blocks, *F*(7, 8) = 1.906, *p = .*098, *η_p_^2^* = 0.192.

Regarding the displayed stimulus, the analysis revealed a lower microsaccade rate for the fixation cross than for the to-be-processed stimulus, *F*(1, 8) = 19.380, *p = .*002, *η_p_^2^* = 0.708. Furthermore, the interaction between stimulus type and displayed object was significant, *F*(1, 8) = 5.438, *p = .*048, *η_p_^2^* = 0.405. Further analyses showed lower microsaccade rate in the letter condition (*M* = 1.71, *SD* ± 0.46) than in the figure condition (*M* = 1.99, *SD* ± 0.57) for the fixation cross, *t*(10) = -5.037, *p* = .001, *η_p_^2^* = .85, but no significant difference for the to-be-processed stimulus (target or distractor), *t*(10) = -5.037, *p* = .001, *η_p_^2^* = .021 (*M_Letter_* = 2.80, *SD* ± 0.47; *M_Figure_* = 2.80, *SD* ± 0.33). All other effects were not significant, all *F*s < 2, *p* < .05.

**Figure 6.**
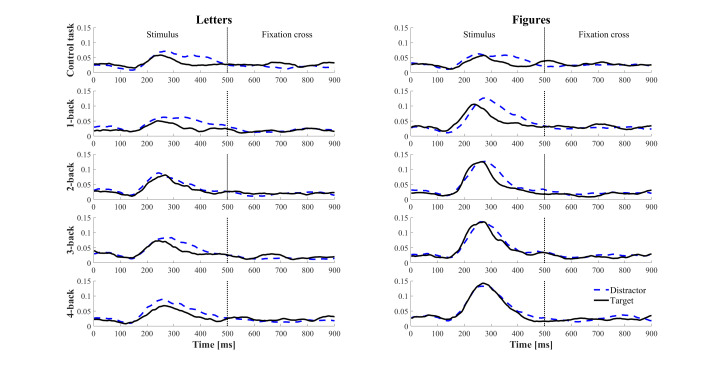
Time evolution of microsaccade rate as a function of stimuli type (visual load) and n-back level (mental load). The blue dashed line represents the microsaccade rate for the distractors and the solid black line the microsaccade rate for the target stimuli (which required an answer from the participants).

The visual inspection of the temporal evolution of microsaccade rate showed a single peak around 270ms after the onset of the to-be-processed stimulus (see Figure 6). Overall, we observed a similar pattern on microsaccade rate as revealed by the 2x5 ANOVA. Regarding the visual load, a higher peak was observed in the figure condition than in the letter condition. The peak amplitude was also affected by the task type, where higher microsaccade rates were showed in the n-back task than in the control task. Furthermore, there was no noticeable difference across n-back levels for both letter and figure conditions. Finally, the onset of target stimuli generated a similar microsaccade rate as distractors. This suggests that microsaccadic response was modulated by the displayed stimuli but not by differences in required stimulus response (distractor vs target).

## Discussion

The aim of this study was to investigate the influence of visual and mental task demand on microsaccade rate in visual tasks. Results showed that microsaccade rate is linked with visual load but not with mental load per se. Microsaccade rate increased from the control condition (probe detection task) to the onset of the n-back figure task (rather simple mental task but visually complex).

Also, microsaccade rate was higher in the figure condition, which induced more visual load, compared to the letter condition and the control condition. The visual analysis of the temporal evolution of microsaccade rate confirmed that a higher peak amplitude was observed in the figure condition compared to the letter condition. Therefore, we can confirm our first hypothesis, stating that we expect higher microsaccade rate in the n-back figure condition compared to the letter condition. These findings are in line with previous studies (9, 11) which found that visual load in a non-exclusive visual task leads to an increase in microsaccade rate. Interestingly, the increase in mental demand did not lead to a significant change in microsaccade rate. Since the manipulation check was successful (i.e. subjective evaluation of task difficulty increased with increasing n-back level), this implies that we have to reject our second hypothesis, stating that microsaccade rate decreases with increasing mental load. These findings contradict previous studies which argue that tasks with high mental demand reduce microsaccade rate (6, 7, 8). Therefore, our study challenges the assumption that microsaccade rate could be a measure for mental workload in tasks which require a certain amount of visual information processing. In our study, we tied the increase in mental demand to a visual task. In all five difficulty levels, participants were asked to identify a letter or a figure and therefore, had to process the visual stimuli to complete the mental task. Therefore, we argue that the amount of required visual processing resources stays the same over the five difficulty levels of the n-back task, which would explain why microsaccade did not decrease despite the increase in mental load.

It could be assumed that in studies showing a decrease in microsaccade rate in tasks of high mental task demand, participants attributed less resources to their visual system and concentrated on solving the demanding mental tasks. In the previous studies showing a decrease of microsaccade rate, mental load was induced through tasks with no visual component whatsoever (e.g. a mental arithmetic task). This resource reduction on visual processes might have led to the decrease in microsaccade rate. In the present study, a minimal amount of visual processing is required in all levels of mental load and hence this could not result in a resource deallocation, suggesting that microsaccade rate is solely linked with visual load and not with mental load.

In comparison to Krueger et al. (9), our mental load condition required some degree of visual information processing and visual load was induced through a task demanding some mental information processing. In Krueger et al. (9) the mental load was induced trough a mental arithmetic task requiring no visual information processing and the visual load was induced through a visual task requiring some mental information processing. Krueger et al. (9) found that the increase in visual load lead to an increase in microsaccade rate. However, in combination with a difficult mental arithmetic task, this increase did not happen. Hence, the conclusion was that the mental arithmetic task pulled away resources from the visual task. However, it would also be possible to argue, that the visual task pulls away resources from the arithmetic task because if presented alone, the arithmetic task lead to a reduction in microsaccade rate.

The results of this study show that mental demand, if bound to a visual task, does not lead to a decrease in microsaccade rate in relation to difficulty increase. Therefore, we conclude that microsaccade rate indeed primarily is influenced by visual load and that a change in microsaccade rate through mental load displays a resource redistribution away from the visual stimuli.

### Task manipulation

In order to exclude potential other influencing factors, we analyzed whether microsaccade rate was modulated by the required action after stimulus presentation (i.e. pushing a button on the keyboard or not). Data analysis revealed no considerable influence of the required action on micorsaccade rate which did not differ between target (requiring action) and distractor stimuli (requiring no action). On the other hand, miccrosaccade rate was increased during stimulus presentation (i.e. target and distractor) compared to the presentation of a fixation cross for both stimuli types (letter and figure). This allows us to conclude that the increase in microsaccade rate observed for figure stimuli (as compared to the letter stimuli) was due to the increase in visual load induced by the figure stimulus type.

### Limitations and future research

In the control task of this experiment, participants were required to identify a predefined stimulus (a star) among distractors (a diamond). However, it would have been more accurate to use letters and Attnaeve figures as control stimuli in this probe detection task. In addition, it could be speculated that differences between stimulus types might be due to the task domain (verbal versus visual-spatial) instead of visual complexity. In a fMRI study however, Ragland and colleagues (38) showed that the same cortical areas are activated during a n-back task using either overlearned (letters) or meaningless stimuli (fractal figures). This suggests that both types of stimuli are processed by the same working memory circuitry. Therefore, the difference in microsaccade rate observed in this study may be primarily attributed to the difference in visual complexity of the stimuli and not to their processing by different working memory systems. While the focus of this piece of research lies in the modulation of microsaccade rate through different task modalities, further research should investigate the link between sustained level of microsaccade rate and event-related modulation (e.g. oculomotor inhibition). In this context, more microsaccades would be expected in focal attention mode of visual inspection compared to ambient attention in a free viewing task (e.g. (39)).

### Implications

The findings presented have several implications for research and practice. Our results support the assumption that in tasks which hold a strong visual component, microsaccade production is increased compared to tasks with no visual component. This implies that a high microsaccade rate (compared to a baseline measurement for the respective person) can indicate if a subject is processing visual information in a task. Microsaccade rate increases in a task if visual load increases. In addition, findings of this study indicate that the decrease of microsaccade rate reported in other studies might not be due to an increase in mental demand as suggested in previous research but be linked with a shift of resources from visual towards mental processes. It remains to be evaluated whether tasks induced by other sensory modalities (e.g. auditory or tactile system) would lead to the same result pattern.

Within an applied context, these findings suggest that visual information processing might be monitored in real time through continuous microsaccade detection. As mentioned by Krueger and colleagues (9), being able to continuously assess the visual attention of a person, for example whether their focus is drifting into inattentional blindness, could be of great value. This could be useful in applied settings where human factors are critical, such as airport baggage screening, air traffic control, radar operating or driving.

### Acknowledgements

We would like to thank Léa Nina Köhnlein for her support in data collection. The publication of this study was funded by the Faculty of Humanities and the Fund for the Support of Open Access Publications of the University of Fribourg.

### Ethics and Conflict of Interest

The author(s) declare(s) that the contents of the article are in agreement with the ethics described in http://biblio.unibe.ch/portale/elibrary/BOP/jemr/ethics.html and that there is no conflict of interest regarding the publication of this paper.
